# Crystal structure of bis­(fluoro­sulfato-κ*O*)xenon(II), Xe(SO_3_F)_2_

**DOI:** 10.1107/S2056989015004788

**Published:** 2015-03-14

**Authors:** Moritz Malischewski, Konrad Seppelt

**Affiliations:** a Freie Universität Berlin, Institut für Chemie und Biochemie – Anorganische Chemie, Fabeckstrasse 34-36, 14195 Berlin, Germany

**Keywords:** crystal structure, xenon, fluoro­sulfate, oxidizer, noble gas, xenon–oxygen compound, superacid

## Abstract

Thermally unstable Xe(SO_3_F)_2_ has been prepared by the reaction of XeF_2_ with HSO_3_F. Single crystals were obtained from HSO_3_F by slow cooling in a sealed tube. The mol­ecular structure is characterized by the Xe atom covalently bonded to two O atoms of two fluoro­sulfate tetra­hedra in an almost linear fashion [O—Xe—O = 179.13 (4)°]. The crystal packing is strongly influenced by inter­molecular van der Waals forces.

## Abstract 2

The Xe atom in Xe(SO_3_F)_2_ is linearly bonded to two O atoms of the fluoro­sulfate unit.

## Chemical context   

In 1972, Neil Bartlett published data on the unit cell of Xe(SO_3_F)_2_ (Wechsberg *et al.*, 1972 ▸). As a result of the thermal instability of this compound, no further structural details were given at that time, but ^19^F and ^129^Xe NMR spectra were reported subsequently (Gillespie *et al.*, 1974 ▸; Schrobilgen *et al.*, 1978 ▸). The decomposition of Xe(SO_3_F)_2_ leads cleanly to Xe and S_2_O_6_F_2_.

## Structural commentary   

**Figure 1:**
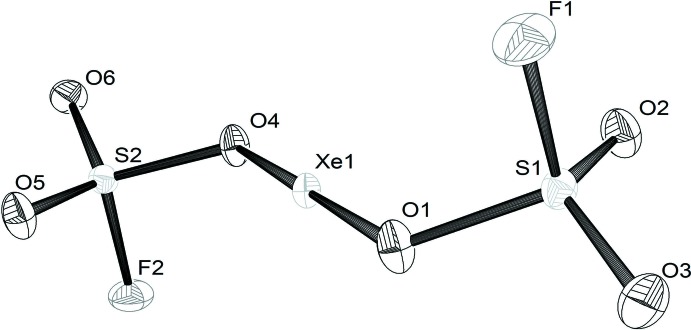
The mol­ecular structure of the title compound, with displacement ellipsoids drawn at the 50% probability level.

Analogous to XeF_2_ (Agron *et al.*, 1963 ▸), the two-coordinated xenon atom adopts a linear geometry [angle O1—Xe—O4 = 179.13 (4)°]. The mol­ecule has nearly *C_i_* symmetry, with the xenon atom at the pseudo-inversion centre (Fig. 1 ▸). This finding is in contrast to earlier reports, where *C_s_* symmetry was discussed based on Raman spectroscopic data (Gillespie & Landa, 1973 ▸). The Xe—O bonds are 2.1101 (13) and 2.1225 (13) Å, which is typical for Xe—O single bonds, whereas Xe=O double bonds are considerably shorter with lengths ≃ 1.75 Å. The related compound xenon fluoride fluoro­sulfate, XeF(OSO_2_F) (Bartlett *et al.*, 1969 ▸, 1972 ▸), contains a Xe—O bond that is slightly longer [2.155 (8) Å] than in the title compound, but the Xe—F bond of XeF(OSO_2_F) is at 1.940 (8) Å shorter than that in XeF_2_ (2.00 Å). For XeF(OSO_2_F), partial ionic bonding (XeF^+^·OSO_2_F^−^) was discussed. Obviously, both XeF_2_ and Xe(SO_3_F)_2_ have a higher covalent character. The S—O bonds in Xe(SO_3_F)_2_ involving the O atoms that are also bonded to the xenon atom (S1—O1 and S2—O4) are about 0.1 Å longer than the terminal S—O bonds (Table 1 ▸), indicating partial double-bond character.

## Supra­molecular features   

**Figure 2:**
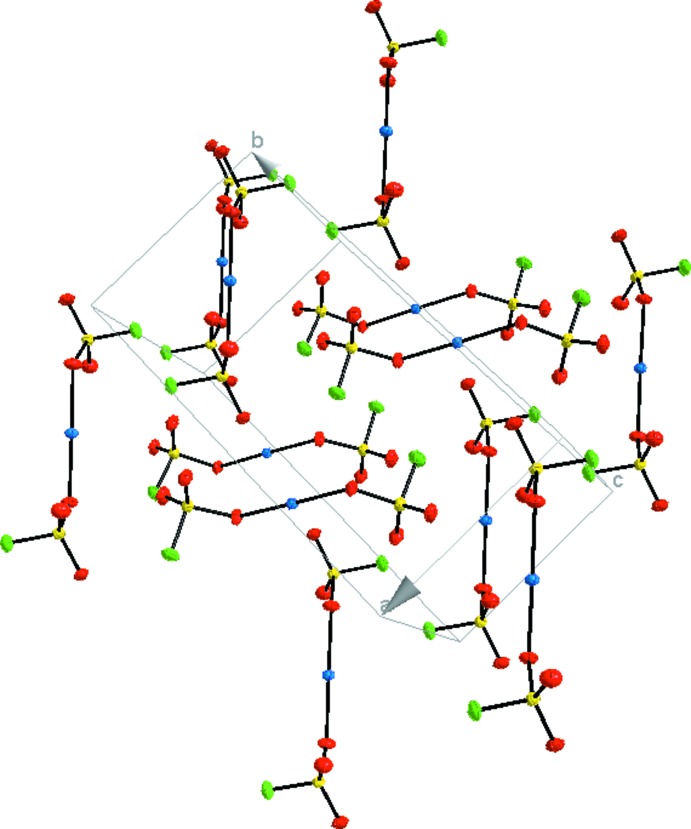
The crystal packing of the title compound

The crystal packing (Fig. 2 ▸) is strongly influenced by inter­molecular van der Waals inter­actions to seven oxygen atoms and two fluorine atoms (Table 2 ▸). Whereas the xenon atom in XeF_2_ exhibits inter­molecular inter­actions to eight fluorine atoms (distance 3.42 Å; Agron *et al.*, 1963 ▸), XeF(OSO_2_F) has fewer contacts (five contacts to oxygen in the range 3.28–3.49 Å and one contact to fluorine of 3.39 Å; Bartlett *et al.*, 1972 ▸).

## Synthesis and crystallization   

550 mg fluoro­sulfuric acid were placed in a 8 mm PFA tube. 170 mg (1 mmol) of XeF_2_ were added and the mixture vigorously shaken at room temperature for some minutes until all XeF_2_ had dissolved. The PFA tube was evacuated for some seconds to remove HF, then frozen with liquid nitro­gen and sealed. The yellow product (≃ 0.2 ml) was warmed to 273 K and the PFA tube placed in a dewar filled with 273 K ethanol and cooled slowly to 193 K in a freezer. The light-yellow single crystals of Xe(SO_3_F)_2_ that had formed were deca­nted off and mounted in a cold nitro­gen stream. At 100 K, the crystals are colorless. The compound decomposes rapidly in moist air and can ignite organic materials.

## Refinement details   

Crystal data, data collection and structure refinement details are summarized in Table 3 ▸.

## Supplementary Material

Crystal structure: contains datablock(s) I. DOI: 10.1107/S2056989015004788/wm5134sup1.cif

Structure factors: contains datablock(s) I. DOI: 10.1107/S2056989015004788/wm5134Isup2.hkl

CCDC reference: 1052852

Additional supporting information:  crystallographic information; 3D view; checkCIF report

## Figures and Tables

**Table 1:** Selected geometric parameters (, )

Xe1O1	2.1101(13)	S1F1	1.5449(12)
Xe1O4	2.1225(13)	S2O6	1.4141(13)
S1O3	1.4103(13)	S2O5	1.4150(14)
S1O2	1.4092(14)	S2O4	1.5237(13)
S1O1	1.5334(13)	S2F2	1.5483(12)
			
O1Xe1O4	179.13(4)		

**Table 2:** Intermolecular contacts ()

Xe1O2	3.1613(15)	Xe1F1^iv^	3.4551(17)
Xe1O5	3.1855(16)	Xe1O3^v^	3.4707(19)
Xe1O2^i^	3.1872(17)	Xe1O5^vi^	3.4818(18)
Xe1O6^ii^	3.2317(19)	Xe1F2^vii^	3.5867(17)
Xe1O6^iii^	3.3262(18)		

Symmetry codes: (i) 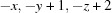; (ii) 
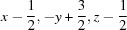
; (iii) 
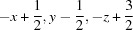
; (iv) 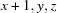; (v) 
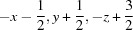
; (vi) 
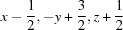
; (vii) 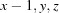.

**Table 3:** Experimental details

Crystal data	
Chemical formula	[Xe(SO_3_F)_2_]
*M*_r_	329.42
Crystal system, space group	Monoclinic, *P*2_1_/*n*
Temperature (K)	100
*a*, *b*, *c* ()	6.706(3), 13.237(6), 7.769(3)
()	96.50(3)
*V* (^3^)	685.2(5)
*Z*	4
Radiation type	Mo *K*
(mm^1^)	5.66
Crystal size (mm)	0.50 0.40 0.15

Data collection	
Diffractometer	Bruker CCD SMART 2000
Absorption correction	Multi-scan (*SADABS*; Bruker, 2006 ▸)
*T*_min_, *T*_max_	0.545, 1.000
No. of measured, independent and observed [*I* > 2(*I*)] reflections	11036, 2096, 1978
*R*_int_	0.020
(sin /)_max_ (^1^)	0.716

Refinement	
*R*[*F* ^2^ > 2(*F* ^2^)], *wR*(*F* ^2^), *S*	0.013, 0.033, 1.11
No. of reflections	2096
No. of parameters	101
_max_, _min_ (e ^3^)	0.56, 0.69

Computer programs: *SMART* and *SAINT* (Bruker, 2006 ▸), *SHELXS97*, *SHELXL* and *SHELXTL* (Sheldrick, 2008 ▸), *ORTEP-3 for Windows* (Farrugia, 2012 ▸) and *DIAMOND* (Brandenburg, 1999 ▸).

## supplementary crystallographic information

### Crystal data

**Table:**

[Xe(SO_3_F)_2_]	*F*(000) = 608
*M**_r_* = 329.42	*D*_x_ = 3.194 Mg m^−^^3^
Monoclinic, *P*2_1_/*n*	Mo *K*α radiation, λ = 0.71073 Å
Hall symbol: -P 2yn	Cell parameters from 999 reflections
*a* = 6.706 (3) Å	θ = 2.0–21.0°
*b* = 13.237 (6) Å	µ = 5.66 mm^−^^1^
*c* = 7.769 (3) Å	*T* = 100 K
β = 96.50 (3)°	Irregular, colorless
*V* = 685.2 (5) Å^3^	0.50 × 0.40 × 0.15 mm
*Z* = 4	

### Data collection

**Table:**

Bruker CCD SMART 2000 diffractometer	2096 independent reflections
Radiation source: fine-focus sealed tube	1978 reflections with *I* > 2σ(*I*)
Graphite monochromator	*R*_int_ = 0.020
ω scans	θ_max_ = 30.6°, θ_min_ = 3.1°
Absorption correction: multi-scan (*SADABS*; Bruker, 2006)	*h* = −9→8
*T*_min_ = 0.545, *T*_max_ = 1.000	*k* = −18→18
11036 measured reflections	*l* = −11→9

### Refinement

**Table:**

Refinement on *F*^2^	Primary atom site location: structure-invariant direct methods
Least-squares matrix: full	Secondary atom site location: difference Fourier map
*R*[*F*^2^ > 2σ(*F*^2^)] = 0.013	*w* = 1/[σ^2^(*F*_o_^2^) + (0.0151*P*)^2^ + 0.4262*P*] where *P* = (*F*_o_^2^ + 2*F*_c_^2^)/3
*wR*(*F*^2^) = 0.033	(Δ/σ)_max_ = 0.002
*S* = 1.11	Δρ_max_ = 0.56 e Å^−^^3^
2096 reflections	Δρ_min_ = −0.69 e Å^−^^3^
101 parameters	Extinction correction: *SHELXL* (Sheldrick, 2008), Fc^*^=kFc[1+0.001xFc^2^λ^3^/sin(2θ)]^-1/4^
0 restraints	Extinction coefficient: 0.0244 (5)

### Special details

**Table:**

Geometry. All s.u.'s (except the s.u. in the dihedral angle between two l.s. planes) are estimated using the full covariance matrix. The cell s.u.'s are taken into account individually in the estimation of s.u.'s in distances, angles and torsion angles; correlations between s.u.'s in cell parameters are only used when they are defined by crystal symmetry. An approximate (isotropic) treatment of cell s.u.'s is used for estimating s.u.'s involving l.s. planes.
Refinement. Refinement of *F*^2^ against ALL reflections. The weighted *R*-factor *wR* and goodness of fit *S* are based on *F*^2^, conventional *R*-factors *R* are based on *F*, with *F* set to zero for negative *F*^2^. The threshold expression of *F*^2^ > 2σ(*F*^2^) is used only for calculating *R*-factors(gt) *etc*. and is not relevant to the choice of reflections for refinement. *R*-factors based on *F*^2^ are statistically about twice as large as those based on *F*, and *R*- factors based on ALL data will be even larger.

### Fractional atomic coordinates and isotropic or equivalent isotropic displacement parameters (Å^2^)

**Table:**

	*x*	*y*	*z*	*U*_iso_*/*U*_eq_	
Xe1	0.000928 (13)	0.627394 (6)	0.726096 (13)	0.01145 (4)	
S1	−0.33404 (6)	0.46260 (3)	0.75371 (5)	0.01295 (8)	
S2	0.32649 (6)	0.79932 (3)	0.70775 (5)	0.01265 (8)	
O4	0.24243 (18)	0.72199 (8)	0.82568 (16)	0.0155 (2)	
F2	0.51324 (16)	0.74159 (8)	0.65972 (16)	0.0227 (2)	
O2	−0.22016 (19)	0.45699 (9)	0.91739 (17)	0.0190 (2)	
F1	−0.52083 (16)	0.52658 (9)	0.78020 (16)	0.0255 (2)	
O6	0.4027 (2)	0.88225 (8)	0.80947 (18)	0.0181 (2)	
O5	0.2017 (2)	0.81316 (9)	0.55006 (17)	0.0202 (3)	
O3	−0.4069 (2)	0.37545 (9)	0.6629 (2)	0.0218 (3)	
O1	−0.23650 (18)	0.53141 (9)	0.62828 (16)	0.0168 (2)	

### Atomic displacement parameters (Å^2^)

**Table:**

	*U*^11^	*U*^22^	*U*^33^	*U*^12^	*U*^13^	*U*^23^
Xe1	0.01151 (6)	0.01094 (6)	0.01174 (7)	−0.00098 (3)	0.00068 (3)	0.00171 (3)
S1	0.01200 (16)	0.01179 (15)	0.0150 (2)	−0.00106 (12)	0.00137 (13)	0.00063 (13)
S2	0.01379 (17)	0.01185 (15)	0.01261 (19)	−0.00147 (12)	0.00274 (13)	−0.00024 (13)
O4	0.0159 (5)	0.0156 (5)	0.0143 (6)	−0.0052 (4)	−0.0010 (4)	0.0026 (4)
F2	0.0194 (5)	0.0234 (5)	0.0268 (6)	0.0023 (4)	0.0093 (4)	−0.0049 (4)
O2	0.0216 (6)	0.0210 (5)	0.0141 (6)	−0.0027 (5)	0.0004 (5)	0.0033 (5)
F1	0.0158 (5)	0.0232 (5)	0.0382 (7)	0.0055 (4)	0.0064 (5)	−0.0005 (5)
O6	0.0221 (6)	0.0147 (5)	0.0181 (7)	−0.0052 (4)	0.0047 (5)	−0.0038 (4)
O5	0.0240 (6)	0.0219 (6)	0.0141 (6)	−0.0038 (5)	−0.0006 (5)	0.0044 (5)
O3	0.0243 (6)	0.0164 (6)	0.0248 (8)	−0.0070 (4)	0.0026 (5)	−0.0041 (5)
O1	0.0167 (5)	0.0191 (5)	0.0139 (6)	−0.0064 (4)	−0.0022 (4)	0.0033 (4)

### Geometric parameters (Å, º)

**Table:**

Xe1—O1	2.1101 (13)	S1—F1	1.5449 (12)
Xe1—O4	2.1225 (13)	S2—O6	1.4141 (13)
S1—O3	1.4103 (13)	S2—O5	1.4150 (14)
S1—O2	1.4092 (14)	S2—O4	1.5237 (13)
S1—O1	1.5334 (13)	S2—F2	1.5483 (12)
			
Xe1···O2	3.1613 (15)	Xe1···F1^iv^	3.4551 (17)
Xe1···O5	3.1855 (16)	Xe1···O3^v^	3.4707 (19)
Xe1···O2^i^	3.1872 (17)	Xe1···O5^vi^	3.4818 (18)
Xe1···O6^ii^	3.2317 (19)	Xe1···F2^vii^	3.5867 (17)
Xe1···O6^iii^	3.3262 (18)		
			
O1—Xe1—O4	179.13 (4)	O6—S2—O4	108.69 (8)
O3—S1—O2	122.00 (8)	O5—S2—O4	112.64 (8)
O3—S1—O1	108.46 (8)	O6—S2—F2	105.47 (8)
O2—S1—O1	112.23 (7)	O5—S2—F2	105.68 (8)
O3—S1—F1	105.94 (8)	O4—S2—F2	100.24 (7)
O2—S1—F1	105.86 (8)	S2—O4—Xe1	119.74 (7)
O1—S1—F1	99.77 (7)	S1—O1—Xe1	119.18 (7)
O6—S2—O5	121.62 (8)		

Symmetry codes: (i) −*x*, −*y*+1, −*z*+2; (ii) *x*−1/2, −*y*+3/2, *z*−1/2; (iii) −*x*+1/2, *y*−1/2, −*z*+3/2; (iv) *x*+1, *y*, *z*; (v) −*x*−1/2, *y*+1/2, −*z*+3/2; (vi) *x*−1/2, −*y*+3/2, *z*+1/2; (vii) *x*−1, *y*, *z*.

## References

[bb1] Agron, P. A., Begun, G. M., Levy, H. A., Mason, A. A., Jones, C. G. & Smith, D. F. (1963). *Science*, **139**, 842–844. 10.1126/science.139.3557.842 17798193

[bb2] Bartlett, N., Wechsberg, M., Jones, G. R. & Burbank, R. D. (1972). *Inorg. Chem.* **11**, 1124–1127.

[bb3] Bartlett, N., Wechsberg, M., Sladky, F. O., Bulliner, P. A., Jones, G. R. & Burbank, R. D. (1969). *J. Chem. Soc. D*, pp. 703–704.

[bb4] Brandenburg, K. (1999). *DIAMOND*. Crystal Impact GbR, Bonn, Germany.

[bb5] Bruker (2006). *SMART*, *SAINT* and *SADABS*. Bruker AXS Inc., Madison, Wisconsin, USA.

[bb6] Farrugia, L. J. (2012). *J. Appl. Cryst.* **45**, 849–854.

[bb7] Gillespie, R. J. & Landa, B. (1973). *Inorg. Chem.* **12**, 1383–1389.

[bb8] Gillespie, R. J., Netzer, A. & Schrobilgen, G. J. (1974). *Inorg. Chem.* **13**, 1455–1459.

[bb9] Schrobilgen, G. J., Holloway, J. H., Granger, P. & Brevard, C. (1978). *Inorg. Chem.* **17**, 980–987.

[bb10] Sheldrick, G. M. (2008). *Acta Cryst.* A**64**, 112–122. 10.1107/S0108767307043930 18156677

[bb11] Wechsberg, M., Bulliner, P. A., Sladky, F. O., Mews, R. & Bartlett, N. (1972). *Inorg. Chem.* **11**, 3063–3070.

